# Long-Term Non-Congenital Cardiac and Renal Complications in Down Syndrome: A Study of 32,936 Patients

**DOI:** 10.3390/children10081351

**Published:** 2023-08-05

**Authors:** Yu-Nan Huang, Jing-Yang Huang, Chung-Hsing Wang, Pen-Hua Su

**Affiliations:** 1 Department of Pediatrics, Chung Shan Medical University Hospital, Taichung 402306, Taiwanchwang5894@gmail.com (C.-H.W.); 2 School of Medicine, Chung Shan Medical University, Taichung 402306, Taiwan; 3 Center for Health Data Science, Chung Shan Medical University Hospital, Taichung 402306, Taiwan; 4 Institute of Medicine, Chung Shan Medical University, Taichung 402306, Taiwan; 5 Division of Genetics and Metabolism, Children’s Hospital of China Medical University, Taichung 404327, Taiwan; 6 School of Medicine, China Medical University, Taichung 404327, Taiwan

**Keywords:** Down syndrome, trisomy 21, renal and urinary tract complications, acute kidney injury, chronic kidney disease, urinary tract complications

## Abstract

Background: Individuals with Down syndrome are at a higher risk of cardiac, renal, and other health issues due to a complex disease physiology. However, few data exist on long-term disease risks to guide prevention and care. We aimed to determine the 10-year incidence of cardiac, renal, and urinary tract complications in Down syndrome versus matched controls. Methods: This retrospective cohort study utilized a large collaborative database. We identified 32,444 patients with Down syndrome and matched controls, excluding those with pre-follow-up target events. Covariates included demographics, lifestyle factors, and comorbidities. Outcomes were ischemic heart disease, hypertension, hypothyroidism, epilepsy, urinary tract infections and chronic kidney disease. We calculated unadjusted and adjusted hazard ratios (HRs) and 95% confidence intervals (CIs) using Cox regression and plotted Kaplan–Meier survival curves. Findings: Over 10 years, Down syndrome patients showed a 3.7-fold higher ischemic heart disease risk (95% CI: 3.0–4.6) and a 1.6-fold higher hypertension risk (95% CI: 1.4–1.8) versus controls. Hypothyroidism (HR = 2.0; 95% CI: 1.7–2.4), epilepsy (HR = 4.5; 95% CI: 3.5–5.8), and urinary tract infection (HR = 3.9; 95% CI: 3.4–4.6) risks were also higher. Chronic kidney disease risk was 2.7-fold greater (95% CI: 2.1–3.5). Survival analysis confirmed a significantly higher incidence of all outcomes in Down syndrome (*p* < 0.0001). Interpretation: This large study found major health challenges in Down syndrome, with risks 3- to 5-fold higher for chronic conditions versus matched controls over 10 years. Though survival remains high with proper care, focusing resources on the prevention and management of complications in this high-risk group can optimize well-being across the lifespan. Future research accounting for limitations here would provide definitive estimates of disease risk in Down syndrome to guide targeted health strategies.

## 1. Introduction

Down syndrome (DS) is the most common chromosomal disorder, affecting 1–2 per 1000 live births and with increasing longevity and prevalence, exposing this population to long-term health risks [1,2]. DS results from triplication of chromosome 21, the most common aneuploidy and characteristic phenotypes accompanying well-described systemic perturbations to metabolism, immunity, hematopoiesis, and endocrinology in trisomy 21 [3,4]. DS patients exhibit several congenital renal abnormalities that increase susceptibility to long-term kidney disease [5]. Compared to age-matched controls, children with DS exhibit significantly smaller kidney size as well as evidence of impaired renal function [6]. While historically thought to be at low risk for kidney disease, research now suggests nephropathy and other renal/urinary tract complications in DS may be under-recognized.

Cardiovascular disease represents a leading cause of morbidity and mortality in those with DS [7,8,9]. The prevalence of cardiovascular disease in individuals with Down syndrome is alarmingly high [10]. This can be attributed to a combination of genetic and lifestyle factors that are often present in this population [11]. The additional chromosome 21 in DS leads to overexpression of certain genes, some of which are involved in the development and progression of cardiovascular disease [12,13,14]. Moreover, individuals with Down syndrome often lead sedentary lifestyles and have dietary habits that further increase their risk of developing cardiovascular disease [11,15]. Congenital heart defects occur in up to 50% of individuals and contribute substantially to poor outcomes [7,9,16]. Additional risk factors include pulmonary hypertension, endocrine and metabolic disorders, and atherosclerosis [7,17,18]. Despite the high risk, cardiovascular disease in Down syndrome is not inevitable [10]. Early detection and intervention can significantly reduce the risk of morbidity and mortality [7]. Disparities in cardiovascular care for those with DS compared to the general public further impact health and longevity but are often overlooked.

Despite recognizing DS confers a risk of certain medical conditions, little research has examined the incidence and interrelation of morbidities, especially renal and urinary tract complications. Understanding health conditions across ages in DS is critical to crafting prevention, treatment, and optimized care. Though at higher risk for health issues, there is a lack of data from DS individuals for lifetime disease risks, patterns, and relationships. Knowledge in this area is key to health promotion, interventions, and improved care for those with DS. Given gaps around nephropathy and other conditions in DS, clarity on age-related morbidities would enable targeted guidelines by age group.

Case reports represent the majority of existing data on kidney and urinary tract disease in Down syndrome, with only 1 to 69 patients described in each study [1,5,19,20,21]. The field of medical research has seen a surge of interest in recent years regarding heart-related outcomes in individuals with Down syndrome [7,8,17]. This trend reflects the growing recognition of the unique health challenges faced by this population and the need for targeted strategies to improve their cardiovascular health. We accessed a large global database to gain a broader and more representative understanding of these complications in Down syndrome over an extended period.

Our 10-year analysis of over 32,000 Down syndrome patients provides a far larger view of renal and urinary tract outcomes than previously possible through limited single-center reports. With most research confined to small case series and studies spanning shorter timeframes, little clarity exists around lifetime risks, disease evolution, and prognosis in this population. This study aimed to determine the 10-year incidence of cardiac, renal, and urinary tract complications in Down syndrome versus matched controls.

## 2. Methods

Outcomes were ischemic heart disease, hypertension, hypothyroidism, epilepsy, urinary tract infections and chronic kidney disease. Exposures were having Down syndrome. Covariates included demographics, lifestyle factors, and comorbidities. Data derivation proceeded via the TriNetX Analytics Platform, a web-based database compiling deidentified electronic health records from over 130 million patients within the Global Collaborative Network. The MetroHealth System institutional review board conferred non-human participant research exempt status upon this study. Searches transpired from 10 June to 10 July 2023. The study population comprised patient cohorts, including patients with Down syndrome manifesting any of the delineated phenotypes, and the control group comprised encounters for examination of eyes and vision without abnormal findings. Phenotypes were defined as described as follows: ICD-10 codes cover conditions including congenital anomalies (Q21.3, Q75.4), hearing/vision loss (H90.3, H25, H26), pituitary/thyroid disorders (E23.0, E03, E34), intellectual disabilities (F70-F73), motor/speech delays (F82, F80.9), sleep apnea/dysphagia (G47.33, R13.10), asthma/seizures (J45, G40), osteoporosis/fractures (M81, M80, M89), malignancies (C00-D49), hypertension/high cholesterol (I10, E78.0), dementia (F03), CVD (I60-I69, I73, I82), respiratory disease (J98), kidney disease (N17-N19), and gastrointestinal disorders (K50-K52). Cohort’s demographics are in Table 1. To safeguard protected health information, TriNetX employs techniques to obfuscate precise patient counts, rounding values to the nearest ten in all analyses. Topaloglu et al. [22] furnish a meticulous depiction of the database, obtainable at https://trinetx.com/companyoverview/ (accessed on 1 June 2023). TriNetX and its constituent healthcare networks demonstrate compliance with the Health Insurance Portability and Accountability Act (HIPAA), upholding rigorous standards for healthcare data privacy and security. Aggregated or individual-level data derived from TriNetX undergo thorough de-identification per HIPAA §164.514 (a) prior to release. Rigorous data quality assessments guarantee adherence to institutional review board requirements and verify data completeness, plausibility, and conformance [22,23]. Anonymity of patients and healthcare organizations as data provenances remains sacrosanct. This study’s conduct aligned with the Declaration of Helsinki’s ethical principles.

### 2.1. Covariate Factors

To rectify baseline inter-cohort discrepancies between Down syndrome and control groups, we deployed the following covariates. We retrospectively examined records 1 year antecedent to the index date to ascertain foundational factors. Propensity score matching furnished control cohorts boasting comparable baseline traits, militating potential bias. Demographic variables encompassed age, sex, ethnicity, and socioeconomic challenges (e.g., employment/unemployment issues (Z56), education/literacy deficits (Z55), occupational hazards (Z57), and housing/financial hardships (Z59)). Lifestyle factors incorporated smoking (Z72.0/F17) and alcohol consumption (K70). Comorbid conditions included type 2 diabetes mellitus (E11), vitamin D deficiency (E55), hyperlipidemia (E78.5), major depressive disorder (F32), sleep disorders (G47), and psychoactive substance misuse (F10-F19). Laboratory measures consisted of blood glucose (≥128 mg/dL), hemoglobin (≥12 g/dL), obesity (BMI ≥ 30 kg/m^2^), glycosylated hemoglobin (≥7%), C-reactive protein (≥40 mg/dL), circulatory triglycerides, cholesterol, low-density lipoprotein, and high-density lipoprotein. Table 1 delineates missing data.

### 2.2. Study Outcomes

We aimed to assess long-term renal and urinary tract complications of DS with 10-year follow-up periods. Outcomes included recurrent urinary tract infections (N39.0), vesicoureteral reflux (N13.70), neurogenic bladder (due to low muscle tone) (N31.9), posterior urethral valves (Q64.2), renal anomalies (like pelviureteric junction obstruction) (Q62.1), congenital malformations of the urinary system (Q60–Q64), glomerulonephritis (N00-N08), renal injury (N17, N18, and N19), nephrotic syndrome (N04), unspecified nephritic syndrome (N05), kidney stones/nephrolithiasis (N20), hypertensive chronic kidney disease (I12), congenital heart defects (Q20–Q28), heart failure (I50), pulmonary hypertension (I27.0), valvular heart disease (I05–I09), coronary artery disease (I20–I25), atherosclerosis (I70), hypertension (I10–I16), arrhythmias (I47), cardiomyopathy (I42), and cardiac arrest (I46). Additional codes for relevant DS conditions like epilepsy (G40), hypothyroidism (E03), intellectual disability (F70, F71, F72, and F73), etc., impact kidney and urinary tract health and validate the reliability of the cohort. The key ICD-10 diagnosis criteria for ischemic heart disease, hypertension, and hypothyroidism: ischemic heart disease (I20–I25), angina pectoris (I20), acute myocardial infarction (I21), subsequent myocardial infarction (I22), other acute ischemic heart diseases (I24), and chronic ischemic heart disease (I25). Diagnosis is based on any of the following: clinical presentation, ECG changes, cardiac biomarker elevations, and angiographic evidence. Hypertension (I10–I15): essential (primary) hypertension (I10), hypertensive heart disease (I11), hypertensive chronic kidney disease (I12), hypertensive heart and chronic kidney disease (I13), and secondary hypertension (I15). Diagnosis is based on persistently elevated BP > 130/80 mmHg on at least 2 occasions. Hypothyroidism (E03): Diagnosis is based on any of the following: low serum-free T4, elevated TSH, and clinical symptoms/signs such as fatigue, weight gain, dry skin, hair loss, cold intolerance, constipation, and bradycardia.

### 2.3. Statistical Analyses

All statistical analyses were performed with TriNetX. We conducted descriptive analyses of the data. We calculated risks of selected long-term cardiac and renal complications as the number of patients with events during follow-up. Patients with Down syndrome contributed person time to 10-year cohorts from their index date. We excluded those who developed cardiac or renal conditions of interest at diagnosis. We plotted Kaplan–Meier curves depicting cumulative incidence of cardiac and renal complications in the Down syndrome cohort. TriNetX obscures counts < 10 to protect health information by rounding to the nearest 10; we noted whenever this occurred.

To derive cohorts boasting equivalence in baseline traits, we enlisted propensity score matching techniques. Specifically, the proprietary algorithm integral to the TriNetX platform enacted one-to-one pairings of Down syndrome and control subjects. This computational matching process integrated multiple parameters, including age, sex, ethnicity, adverse socioeconomic factors (e.g., housing insecurity, unemployment, educational handicaps, occupational hazards), and lifestyle behaviors (namely smoking and alcohol consumption). Standardized mean differences (SMDs) evaluated post-match balance, with SMD < 0.1 indicating adequate balance. Cox models calculated HRs and 95% confidence intervals (CIs), testing proportionality using the generalized Schoenfeld approach. Kaplan–Meier curves depicted survival probabilities. Two-sided *p* < 0.05 indicated statistical significance. Reporting followed STROBE recommendations.

## 3. Results

### 3.1. Baseline Characteristics

**Figure 1:**
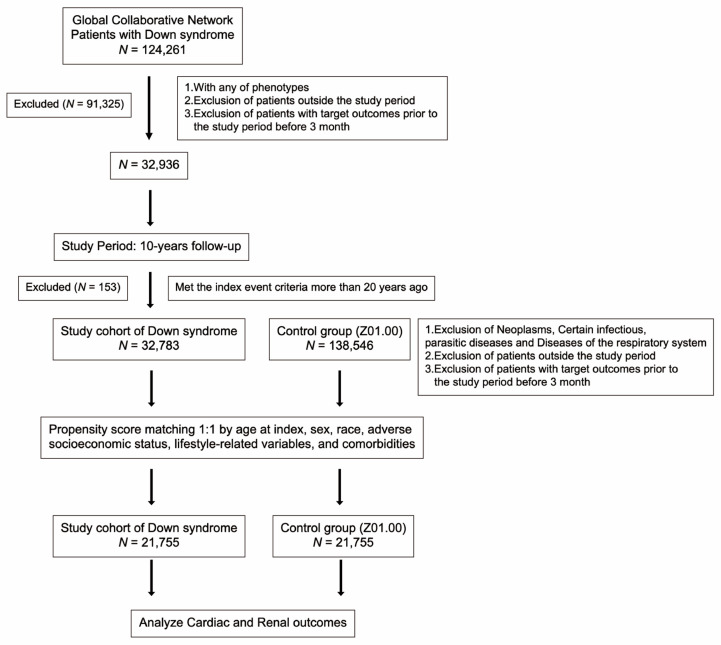
Flowchart of compare cohort construction.

The flow diagram outlines how we constructed the cohort for this study of DS (Figure 1). We began with 124,261 DS patients from the Global Collaborative Network. We first excluded 91,325 patients based on the following: not having any of the phenotypes, being outside the study period, or having target outcomes within 3 months before the study (32,936 remaining). The 10-year study period began here. We excluded 492 patients meeting index event criteria > 20 years ago (32,444 remaining). This rigorous selection left 32,444 DS patients monitored for cardiac, renal, and urinary tract outcomes as our study focused on long-term cardiac, renal, and urinary tract complications.

The comprehensive baseline characteristics of our 32,444 patients with Down syndrome, shown in Table 1 and Table 2, provide a critical context for examining 10-year risks of cardiac, renal, and urinary tract disease in this group. Most were female (72%) and white (68%), with a mean age of 24.5 years. Strikingly, the mean body mass index (BMI) (28.8 kg/m^2^) fell within the overweight range, indicating a need for weight and risk management strategies. Mean systolic (116 mmHg) and diastolic (73.2 mmHg) blood pressure, available for 27%, were normal, suggesting hypertension may not broadly impact this population. Renal function, assessed using mean creatinine (0.915 mg/dL), was normal, but the below-normal mean calcium level (8.98 mg/dL) could indicate underlying bone disease necessitating follow-up. The metabolic profile, including mean cholesterol (176 mg/dL) and glucose (93.3 mg/dL) levels, was largely favorable, comparable to the general public, indicating good control. Liver enzymes and blood counts were typically normal. However, the below-normal mean iron level (67.1 μg/dL) may reflect deficiency from intestinal or absorption issues in Down syndrome, and the elevated mean ferritin (176 ng/mL) could signify disease severity and chronic inflammation common in Down syndrome. In summary, though typically overweight, this cohort exhibited largely normal health parameters. However, calcium/bone and iron regulation issues may be concerns, and elevated ferritin could reflect complex disease physiology in Down syndrome. These baseline insights set the stage to determine the risks of long-term disease, aiming to curb complications through proactive monitoring and treatment in this medically complex group.

**Figure A1:**
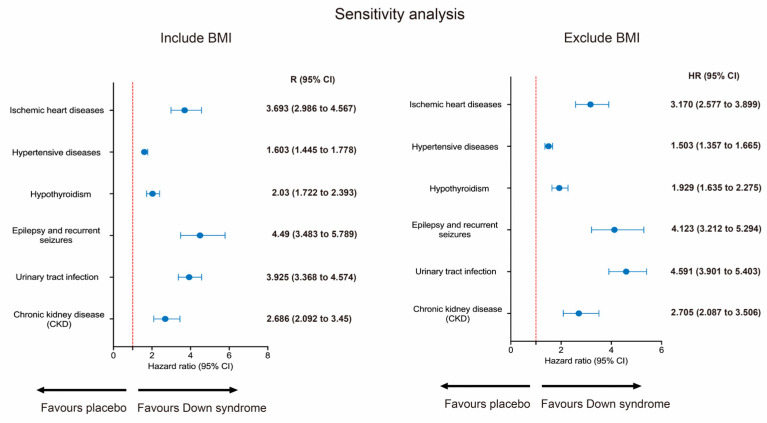
Sensitivity analysis for incorporation or exclusion of BMI.

This propensity score-matched analysis demonstrates notable differences in baseline health characteristics between adults with Down syndrome compared to matched controls without Down syndrome. After propensity score matching on demographic variables, including age, sex, and ethnicity, the cohort with Down syndrome (*n* = 21,755) was well balanced to the control cohort (*n* = 21,755) on these parameters (Table 2). The mean age in the Down syndrome cohort was 23.4 years (standard deviation 17.2 years). Females comprised 60.7% of the Down syndrome cohort, and the predominant ethnicity was White (60.9%). Despite balancing for demographics, adults with Down syndrome exhibited significantly higher rates of several comorbidities compared to matched controls, including type 2 diabetes mellitus (3.3% vs. 3.6%, *p* < 0.001), depression (6.2% vs. 6.3%, *p* = 0.580), and sleep disorders (7.7% vs. 8.4%, *p* = 0.016). Additionally, the Down syndrome cohort demonstrated a proinflammatory state not present in matched controls, with higher mean body mass index (BMI) (26.0 vs. 23.5 kg/m^2^, *p* < 0.001) and C-reactive protein (CRP) levels (29.3 vs. 14.5 mg/L, *p* < 0.001). The high amount of missing lab data is a limitation. We therefore conducted sensitivity analyses excluding BMI, and the final results of these analyses were consistent with the initial report (Figure A1).

### 3.2. Incidence of Long-Term Cardiac and Renal Complications in the Down Syndrome and Control Groups

**Figure 2:**
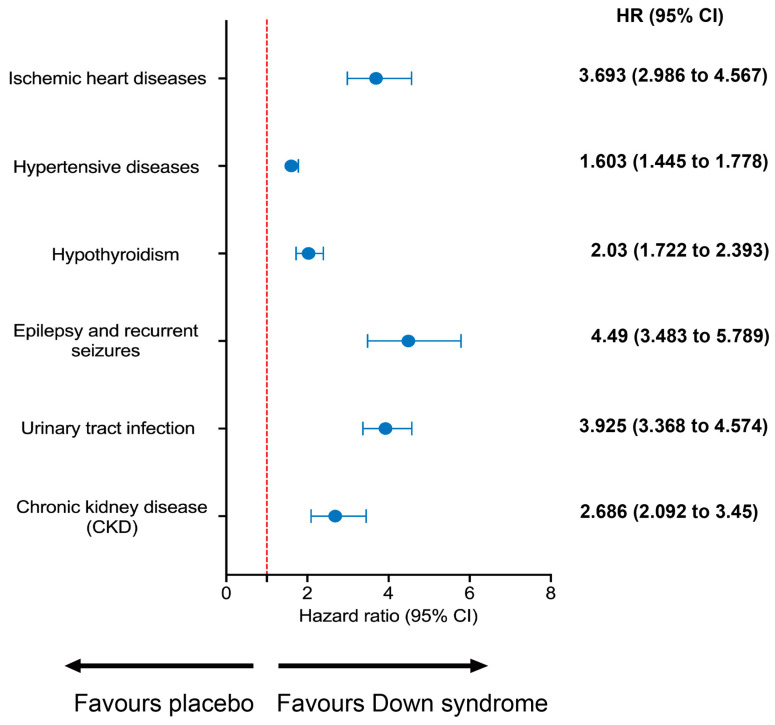
Risk of cardiac and renal complications compared with the control cohort. Prior to commencement of the investigational window, 174 Down syndrome patients and 160 control patients evincing ischemic heart disease were omitted from the analytic dataset, given their antecedent acquisition of the outcome of interest. Similarly, 917 Down syndrome and 977 control patients exhibiting preexisting hypertension were excluded, as were 1010 Down syndrome and 252 control patients with prior hypothyroidism. Likewise, the data excluded 364 Down syndrome and 128 control patients with pre-study epilepsy and seizures, along with 1047 Down syndrome and 287 control patients with earlier urinary tract infections. Finally, 359 Down syndrome and 82 control patients demonstrating chronic kidney disease preceding the observational period were also withheld from the final cohort, precluding contamination of the results by pre-established disease.

Our decade-long observational study assessed risks of long-term cardiac and renal complications in Down syndrome versus controls (Figure 2). We found substantially higher risks across conditions in Down syndrome. Down syndrome showed 3.693 times the ischemic heart disease risk of controls (95% CI: 2.986–4.567). Hypertensive disease risk was also 1.603 times higher (95% CI: 1.445–1.778). Hypothyroidism risk was 2.03 times greater (95% CI: 1.722–2.393). Epilepsy/seizure risk was 4.49 times higher in Down syndrome (95% CI: 3.483–5.789). Urinary tract infection risk was 3.925 times greater (95% CI: 3.368–4.574). Chronic kidney disease risk was 2.686 times higher (95% CI: 2.092–3.45). This rigorous analysis shows major health challenges in Down syndrome, underscoring the need for proactive, targeted health strategies.

### 3.3. Kaplan–Meier Survival Analysis

**Figure 3:**
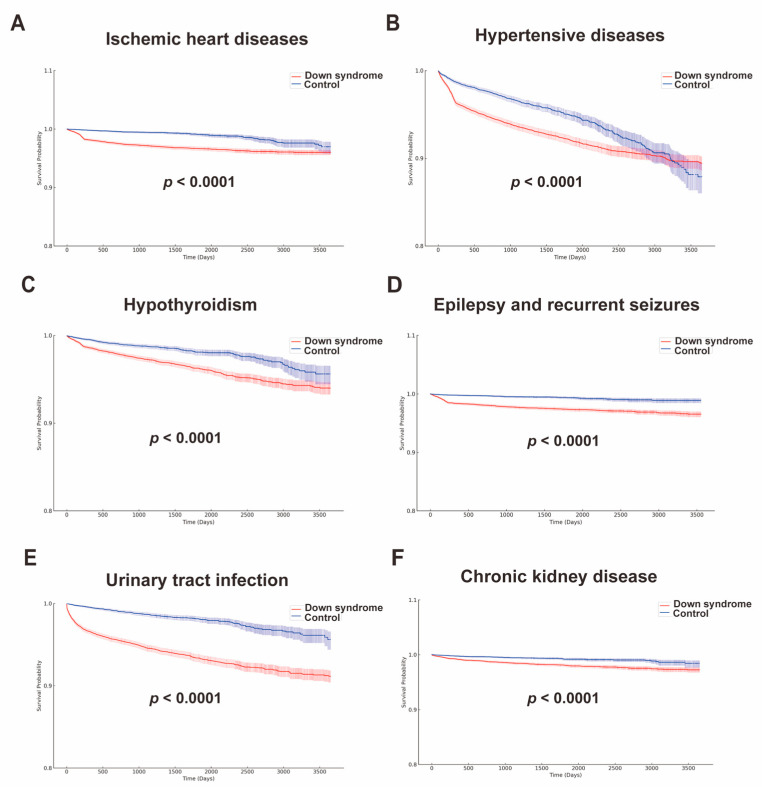
Kaplan–Meier curves of cardiac and renal complications. (**A**) Ischemic heart disease; (**B**) hypertension; (**C**) hypothyroidism; (**D**) epilepsy and seizures; (**E**) urinary tract infections; and (**F**) chronic kidney disease.

Kaplan–Meier survival analysis revealed a significantly higher 10-year incidence of ischemic heart disease in patients with Down syndrome compared to matched controls. The survival curves diverged immediately, indicating differing experiences from the outset (*p* < 0.0001; Figure 3A). Control group survival remained superior throughout follow-up, with final survival of 96.01% for Down syndrome and 96.97% for controls. We excluded those with pre-follow-up events (Down syndrome: *n* = 174; control: *n* = 160). The hypertension outcomes between Down syndrome (*n* = 20,838) and control (*n* = 20,778) groups revealed a substantial survival disparity. In Down syndrome, 1143 (5.5%) had outcomes, with 89.44% survival. Controls had better outcomes (*n* = 526; 2.5%) and 87.88% survival (*p* < 0.0001; Figure 3B). Kaplan–Meier analysis revealed a 2.7-fold higher hypothyroidism risk in Down syndrome over 10 years (*n* = 20,745) versus controls (*n* = 21,503). Hypothyroidism developed in 529 (2.6%) with Down syndrome and 195 (0.9%) controls, with 93.99% versus 95.61% survival (*p* < 0.0001; Figure 3C). Longitudinal analysis showed 5.8-fold higher epilepsy/seizure risk over 10 years in Down syndrome (*n* = 21,391) versus controls (*n* = 21,627). Epilepsy developed in 405 (1.9%) with Down syndrome and 70 (0.3%) controls, with 96.44% versus 98.89% survival (*p* < 0.0001; Figure 3D). Down syndrome (*n* = 20,708) also showed a 4.9-fold higher 10-year UTI risk versus controls (*n* = 21,468). UTIs developed in 976 (4.7%) with Down syndrome and 198 (0.9%) controls, with 91.14% versus 95.61% survival (*p* < 0.0001; Figure 3E). Finally, a 3.6-fold higher CKD risk emerged over 10 years in Down syndrome (*n* = 21,396) versus controls (*n* = 21,673). CKD developed in 281 (1.3%) with Down syndrome and 79 (0.4%) controls, with 97.23% versus 98.42% survival (*p* < 0.0001; Figure 3F).

In summary, while Down syndrome survival remains high, this population shows risks for comorbidities that, if managed well, seem largely avoidable, giving reason for cautious optimism.

## 4. Discussion

The key finding was that over 10 years of follow-up, patients with Down syndrome exhibited a substantially higher incidence of chronic diseases, including ischemic heart disease, hypertension, hypothyroidism, epilepsy, urinary tract infections, and chronic kidney disease compared to matched controls. A large longitudinal cohort study provides crucial insights into the long-term health risks associated with Down syndrome.

The 3- to 5-fold greater risks across most conditions highlight the significant medical vulnerabilities in Down syndrome and underscore the need for proactive screening, monitoring, and management in this high-risk group [7,24,25]. While survival remains high with appropriate care, many complications appear preventable or controllable, suggesting an opportunity for optimizing health, function, and quality of life [16,26,27]. These results demand action to curb morbidity through early risk assessment, lifestyle intervention, and treatment of co-existing conditions.

The cohort characteristics provide context for the complications observed. Mean BMI within the overweight range and suboptimal calcium/iron levels indicate avenues for risk reduction. However, largely normal metabolic and hemodynamic parameters suggest good access to health care. The elevated mean ferritin level may signal chronic inflammation, common in Down syndrome, that could underlie certain disease risks [28,29,30].

Individuals with Down syndrome often require medical and surgical interventions to manage associated comorbidities and improve quality of life. Common conditions and their treatments include congenital heart defects, such as atrioventricular septal defects, which are repaired surgically early in life. Gastrointestinal issues like esophageal atresia and Hirschsprung disease may also need surgical corrections. Obstructive sleep apnea is managed with continuous positive airway pressure (CPAP) or surgeries like adenotonsillectomy. Hypothyroidism is treated with thyroid hormone replacement. Leukemia risk is increased, so chemotherapy is utilized if needed. Vision and hearing problems can be corrected with glasses, surgery (e.g., cataract removal), or devices like hearing aids. A small percentage have atlantoaxial instability, requiring surgery to realign the cervical vertebrae. Mental health conditions are managed through counseling and medications. Early-onset Alzheimer’s disease is common and currently treated by managing symptoms. Obesity and related comorbidities are addressed through lifestyle interventions. Other medications used include antibiotics for infections, anticonvulsants for seizures, and supplements to correct nutritional deficiencies.

This study’s major strengths include large sample size, extended follow-up, the importance of finding care for long-term urinary tract infections and renal complications in DS, as well as the use of a matched control group, enabling robust comparisons. However, its retrospective nature may have introduced selection bias. Covariate adjustment likely did not fully account for residual confounding. Loss to follow-up and inadequate capture of outcomes may have led to the underestimation of risks. Lacking individual-level data, the analyses could not adjust for certain factors like disease severity. However, excluding missing data cannot solve the fundamental problem, especially when dealing with missing data that are not missing at random. Even after excluding missing data, the accuracy of the estimates can still be affected [31]. Regarding the issue of missing BMI data, there are no raw data available for multiple imputation. This research relies on electronic health records (EHRs) from the TriNetX network. Such data have inherent limitations that we acknowledge. Residual confounding likely persists, as EHRs incompletely capture social and economic factors that influence outcomes [32]. EHRs do not reveal where diagnoses occurred (primary, secondary, or specialist care) or the diagnosing clinician. Patients may visit multiple healthcare organizations (HCOs), so records can be incomplete if an HCO does not participate in the network. EHRs lack details on diagnosis severity and duration, permitting only analysis of incidence, not illness persistence. Our diagnosis dates may not match when the criteria were first met. Inaccuracies may arise from mis-recorded data. Historical data before EHR adoption or HCO network participation can be incomplete, omitting prior diagnoses or outcomes. Overall, these factors introduce uncertainty. However, EHRs remain vital for real-world evidence generation. This study adds value to the literature while openly acknowledging its limitations. Further research using multi-source data is warranted to validate signals from EHR analyses. Active surveillance and continual validation will allow us to leverage EHR data to advance clinical knowledge.

In summary, this sizeable study shows significantly higher risks of chronic diseases in DS that demand action to promote health through the lifespan. While cautious optimism remains, focusing research and resources on preventing and managing complications can optimize well-being for individuals with DS and ease caregiver burden. In the future, a prospective study accounting for the limitations identified would provide more definitive estimates of disease risk in DS to guide targeted health strategies. Overall, this work highlights the need to make the health of those with DS a priority in order to enhance longevity and quality of life.

## Figures and Tables

**Table 1:** Baseline characteristics of patients with Down Syndrome (DS).

	Mean ± SD	Patients with DS, *N* (% of Cohort)
Sex		
Female		23,421 (72)
Male		9003 (28)
Unknown Gender		20 (0.0)
Race		
White		22,042 (68)
Black or African American		3816 (12)
Asian		918 (3)
American Indian or Alaska Native		170 (1)
Native Hawaiian or Other Pacific Islander		73 (0)
Unknown		5425 (17)
Age at index	24.5 ± 15	32,444 (100)
BMI, kg/m^2^	28.8 ± 7.54	17,667 (57)
Systolic Blood Pressure, mmHg	116 ± 14.6	20,980 (67)
Diastolic Blood Pressure, mmHg	73.2 ± 14.5	20,960 (67)
Cholesterol, mg/dL	176 ± 37.7	4351 (14)
LDL, mg/dL	102 ± 31.7	4199 (13)
HDL, mg/dL	50.8 ± 14.9	4224 (14)
Triglyceride, mg/dL	123 ± 89.9	4295 (14)
Glucose, mg/dL	93.3 ± 67.3	12,228 (39)
Albumin, g/dL	3.96 ± 0.637	9266 (30)
Creatinine, mg/dL	0.915 ± 6.06	10,701 (34)
Calcium, mg/dL	8.98 ± 0.973	9669 (31)
Leukocytes, 10^3^/µL	34.1 ± 160	17,152 (55)
Erythrocytes, 10^6^/µL	4.17 ± 0.662	17,271 (55)
Platelets, 10^3^/µL	276 ± 156	17,480 (56)
Alanine aminotransferase, U/L	28.3 ± 51.6	9794 (31)
Aspartate aminotransferase, U/L	28.9 ± 46.5	9714 (31)
Alkaline phosphatase, U/L	109 ± 84.4	8538 (27)
Iron, µg/dL	67.1 ± 47.1	1730 (6)
Ferritin, ng/mL	176 ± 1471	2159 (7)

Data on the number of patients held in the case database. The number of patients in the filtered cohort are 32,444. Abbreviation: BMI, body mass index; LDL, low density lipoprotein; HDL, high density lipoprotein.

**Table 2:** Baseline characteristics of propensity score-matched Down syndrome cohort.

	Before Matching				After Matching			
	Down Syndrome Cohort (*n* = 32,783)	Control Cohort (*n* = 138,546)	Std Diff.	*p* Value	Down Syndrome Cohort (*n* = 21,755)	Control Cohort (*n* = 21,755)	Std Diff.	*p* Value
Age at index								
Mean ± SD	24.5 ± 15.0	22.3 ± 20.8	0.123	<0.001	23.4 ± 17.2	25.3 ± 20.1	0.104	<0.001
Sex (%)								
Female	23,367 (72.4)	68,208 (50.4)	0.462	<0.001	13,201 (60.7)	12,461 (57.3)	0.069	<0.001
Male	8917 (27.6)	67,044 (49.6)	0.463	<0.001	8544 (39.3)	9285 (42.7)	0.069	<0.001
Missing	10 (0.0)	21 (0.0)	0.010	0.067	10 (0.0)	10 (0.0)	<0.001	1
Race (%)								
White	21,988 (68.1)	64,165 (47.4)	0.428	<0.001	13,258 (60.9)	13,246 (60.9)	0.001	0.906
Black or African American	3817 (11.8)	23,270 (17.2)	0.153	<0.001	2604 (12.0)	2682 (12.3)	0.011	0.252
Asian	879 (2.7)	7149 (5.3)	0.131	<0.001	685 (3.1)	667 (3.1)	0.005	0.619
American Indian	169 (0.5)	822 (0.6)	0.011	0.076	103 (0.5)	88 (0.4)	0.010	0.277
Native Hawaiian	44 (0.1)	283 (0.2)	0.018	0.008	29 (0.1)	38 (0.2)	0.011	0.271
Missing or unknown	5397 (12.6)	39,584 (13.0)	0.302	<0.001	5076 (23.3)	5034 (23.1)	0.005	0.634
Social economic status								
Housing/economic circumstances problem	6526 (20.2)	158 (0.1)	0.705	<0.001	90 (0.4)	133 (0.6)	0.028	0.004
Employment and unemployment problems	691 (2.1)	108 (0.1)	0.198	<0.001	74 (0.3)	81 (0.4)	0.005	0.573
Problems related to education and literacy	267 (0.8)	203 (0.2)	0.097	<0.001	73 (0.3)	79 (0.4)	0.005	0.626
Occupational exposure to risk factors	182 (0.6)	55 (0.0)	0.095	<0.001	30 (0.1)	24 (0.1)	0.008	0.414
Lifestyle								
Tobacco use (smoking)	8909 (27.6)	333 (0.2)	0.860	<0.001	331 (1.5)	327 (1.5)	0.002	0.875
Nicotine dependence (smoking)	4994 (15.5)	1308 (1.0)	0.547	<0.001	675 (3.1)	658 (3.0)	0.005	0.636
Alcohol liver disease (alcohol drinking)	10 (0.0)	24 (0.0)	0.008	0.134	10 (0.0)	12 (0.1)	0.004	0.670
Comorbidities								
Type 2 diabetes mellitus	3770 (11.7)	3695 (2.7)	0.351	<0.001	725 (3.3)	783 (3.6)	0.015	0.128
Vitamin D deficiency	702 (2.2)	1521 (1.1)	0.082	<0.001	443 (2.0)	554 (2.5)	0.034	<0.001
Hyperlipidemia	545 (1.7)	2296 (1.7)	0.001	0.904	421 (1.9)	554 (2.5)	0.041	<0.001
Depression	7649 (23.7)	2381 (1.8)	0.697	<0.001	1353 (6.2)	1381 (6.3)	0.005	0.580
Sleep disorder	5688 (17.6)	2081 (1.5)	0.568	<0.001	1682 (7.7)	1819 (8.4)	0.023	0.016
Psychoactive substance use	7627 (23.6)	1993 (1.5)	0.709	<0.001	1090 (5.0)	1099 (5.1)	0.002	0.844
Laboratory								
BMI								
*n* (%)	16,756 (51.9)	22,749 (16.8)	0.784	<0.001	6992 (32.1)	5818 (26.7)	0.338	<0.001
mean ± SD, kg/m2	27.5 ± 7.3	22.0 ± 6.7	0.784	<0.001	26.0 ± 7.9	23.5 ± 6.9	0.338	<0.001
≥30 kg/m^2^, *n* (%)	3637 (11.1)	2359 (1.7)	0.595	<0.001	1505 (6.9)	671 (3.1)	0.036	<0.001
Missing	12,390 (37.8)	115,797 (83.6)			14,763 (67.9)	15,937 (73.3)		
CRP								
*n* (%)	1061 (3.3)	1580 (1.2)			737 (3.4)	840 (3.9)		
mean ± SD, mg/L	26.4 ± 45.9	11.8 ± 31.8	0.371	<0.001	29.3 ± 47.8	14.5 ± 36.8	0.349	<0.001
Missing	1061 (3.3)	1581 (1.2)	0.144	<0.001	737 (3.4)	841 (3.9)	0.026	0.008

BMI: Body mass index; Std diff: standardized difference; CRP: C reactive protein; If the number of patients is less than or equal to 10, the result is displayed as 10 for privacy protection purposes.

## Data Availability

Data are available upon https://trinetx.com/companyoverview/.
